# Supplementary material for: Comparison of Intensive Care Scoring Systems in Predicting Overall Mortality of Sepsis
Source: Diagnostics (Basel). 2025 Jun 29;15(13):1660. doi: 10.3390/diagnostics15131660 (PMC12248853; doi:10.3390/diagnostics15131660)
Supplement: Supplementary file 1 [file diagnostics-15-01660-s001.zip › diagnostics-3488191-supplementary.pdf]

Supplementary Table S1: Patient Scoring Results and Demographic Information

ID	Gender	Age	Charlson Comorbidity Index	APACHE II	SOFA	SAPS II	OASIS	28 Days Mortality	Ionotrop Support Requirement	Mechanical Ventilation Requirement
1	Male	61	6	25	13	59	41	Non-survivor	Required	Not Required
2	Male	66	11	37	13	74	35	Non-survivor	Required	Required
3	Male	63	4	44	19	67	48	Non-survivor	Required	Required
4	Male	78	7	14	5	36	21	Survivor	Not Required	Required
5	Female	67	4	23	6	42	24	Survivor	Not Required	Required
6	Male	76	8	34	8	51	38	Non-survivor	Required	Not Required
7	Male	68	4	26	8	53	31	Non-survivor	Not Required	Not Required
8	Male	70	12	29	8	67	41	Non-survivor	Not Required	Required
9	Male	59	9	32	10	56	37	Non-survivor	Not Required	Not Required
10	Male	75	7	48	12	61	33	Non-survivor	Required	Required
11	Male	47	4	27	8	44	34	Survivor	Required	Required
12	Female	18	3	25	4	26	18	Survivor	Not Required	Required
13	Female	83	6	32	6	44	39	Non-survivor	Required	Not Required
14	Male	74	7	20	5	53	24	Survivor	Not Required	Required
15	Female	82	7	23	7	62	41	Survivor	Not Required	Not Required
16	Female	82	9	22	7	52	30	Survivor	Not Required	Required
17	Male	76	5	13	5	44	21	Survivor	Not Required	Required
18	Male	69	10	30	8	77	46	Non-survivor	Required	Required
19	Female	68	5	23	5	39	22	Survivor	Not Required	Required
20	Female	90	8	21	7	62	28	Non-survivor	Not Required	Required
21	Male	61	4	11	4	39	19	Survivor	Not Required	Required
22	Female	65	4	19	6	39	28	Survivor	Not Required	Required
23	Male	90	7	21	6	43	22	Non-survivor	Not Required	Required
24	Male	68	10	33	9	52	46	Non-survivor	Not Required	Not Required
25	Male	87	6	24	7	90	48	Non-survivor	Required	Required
26	Female	82	6	27	8	45	37	Survivor	Required	Required
27	Female	86	6	33	12	53	41	Non-survivor	Not Required	Required
28	Male	68	6	19	6	53	39	Non-survivor	Required	Required
29	Male	60	6	26	4	49	28	Survivor	Not Required	Required
30	Female	65	4	19	6	43	33	Survivor	Not Required	Required
31	Female	92	7	22	5	42	34	Survivor	Not Required	Required
32	Male	76	7	23	8	63	37	Non-survivor	Required	Required
33	Male	68	4	14	7	61	47	Non-survivor	Required	Not Required
34	Male	77	11	33	10	66	47	Non-survivor	Not Required	Required
35	Male	61	4	19	5	33	22	Survivor	Not Required	Required
36	Male	67	4	18	4	65	32	Non-survivor	Required	Required
37	Male	63	6	22	4	47	19	Survivor	Not Required	Required
38	Male	90	7	22	12	52	34	Non-survivor	Required	Required
39	Male	65	4	27	5	36	33	Survivor	Not Required	Required
40	Female	54	5	32	7	56	35	Non-survivor	Required	Required
41	Female	84	8	33	9	67	36	Non-survivor	Required	Required

42	Female	86	13	13	10	68	38	Non-survivor	Required	Required
43	Male	88	6	31	8	53	39	Non-survivor	Not Required	Required
44	Male	82	10	38	11	43	39	Non-survivor	Not Required	Required
45	Female	80	6	11	4	44	25	Survivor	Not Required	Required
46	Male	88	6	29	10	57	45	Non-survivor	Not Required	Required
47	Male	60	4	18	6	48	36	Survivor	Not Required	Required
48	Male	53	5	37	14	54	35	Non-survivor	Required	Not Required
49	Male	62	7	36	10	66	36	Non-survivor	Not Required	Required
50	Female	92	7	28	9	74	33	Non-survivor	Required	Required
51	Female	23	2	21	6	12	13	Survivor	Not Required	Required
52	Female	78	8	26	7	53	34	Non-survivor	Required	Not Required
53	Male	81	12	35	11	78	45	Non-survivor	Required	Required
54	Male	83	6	22	5	62	32	Non-survivor	Not Required	Required
55	Male	66	10	39	16	73	22	Non-survivor	Not Required	Required
56	Male	87	10	41	15	76	34	Non-survivor	Required	Required
57	Male	66	10	19	6	65	39	Non-survivor	Required	Required
58	Female	88	6	25	5	65	32	Survivor	Required	Required
59	Male	58	5	19	5	56	22	Survivor	Not Required	Required
60	Male	62	6	29	9	63	45	Non-survivor	Required	Required
61	Male	69	8	40	12	55	31	Non-survivor	Not Required	Not Required
62	Male	85	11	31	10	66	38	Non-survivor	Required	Required
63	Male	78	7	39	13	53	41	Non-survivor	Not Required	Required
64	Male	70	7	23	7	63	50	Non-survivor	Not Required	Required
65	Female	92	10	20	7	74	52	Non-survivor	Required	Required
66	Female	87	10	28	6	60	36	Non-survivor	Required	Required
67	Female	84	9	38	11	60	47	Non-survivor	Required	Required
68	Male	85	12	31	10	81	53	Non-survivor	Required	Not Required
69	Male	70	5	22	5	38	21	Survivor	Not Required	Required
70	Female	39	8	35	9	65	41	Non-survivor	Required	Required
71	Male	63	4	22	6	48	21	Survivor	Not Required	Required
72	Male	56	4	19	9	51	36	Non-survivor	Not Required	Not Required
73	Male	72	8	17	8	55	25	Survivor	Not Required	Required
74	Female	55	5	23	8	48	40	Survivor	Not Required	Required
75	Male	74	7	36	10	59	26	Non-survivor	Not Required	Required
76	Male	48	2	23	7	49	28	Survivor	Not Required	Required
77	Female	78	10	18	7	78	44	Non-survivor	Required	Required
78	Male	80	7	24	7	49	26	Non-survivor	Not Required	Required
79	Male	62	10	21	5	74	39	Non-survivor	Required	Not Required
80	Female	79	13	33	12	72	51	Non-survivor	Required	Required
81	Male	26	2	14	5	21	10	Survivor	Not Required	Required
82	Male	27	3	26	8	35	32	Survivor	Not Required	Required
83	Female	87	10	28	6	65	42	Non-survivor	Required	Required
84	Male	61	6	25	13	64	44	Non-survivor	Required	Not Required
85	Male	66	11	37	13	71	40	Non-survivor	Required	Required
86	Male	63	4	44	19	63	43	Non-survivor	Required	Not Required

87	Male	78	7	14	5	50	30	Survivor	Not Required	Required
88	Female	67	4	23	6	56	24	Survivor	Not Required	Required
89	Male	76	8	34	8	67	37	Non-survivor	Required	Required
90	Male	68	4	26	8	70	39	Non-survivor	Not Required	Not Required
91	Male	70	12	29	8	66	34	Non-survivor	Not Required	Not Required
92	Male	59	9	32	10	59	41	Non-survivor	Not Required	Required
93	Male	75	7	48	12	86	51	Non-survivor	Required	Required
94	Male	47	4	27	8	83	47	Survivor	Required	Required
95	Female	18	3	25	4	27	15	Survivor	Not Required	Required
96	Female	83	6	32	6	56	53	Non-survivor	Required	Required
97	Male	74	7	20	5	47	22	Survivor	Not Required	Not Required
98	Female	82	7	23	7	50	42	Survivor	Not Required	Required
99	Female	82	9	22	7	52	25	Survivor	Not Required	Required
100	Male	76	5	13	5	40	24	Survivor	Not Required	Required
101	Male	69	10	30	8	65	51	Non-survivor	Required	Required
102	Female	68	5	23	5	35	25	Survivor	Not Required	Required
103	Female	90	8	21	7	52	26	Non-survivor	Not Required	Required
104	Male	61	4	11	4	36	24	Survivor	Not Required	Required
105	Female	65	4	19	6	36	24	Survivor	Not Required	Required
106	Male	90	7	21	6	39	24	Non-survivor	Not Required	Required
107	Male	68	10	33	9	79	47	Non-survivor	Not Required	Required
108	Male	87	6	24	7	65	48	Non-survivor	Required	Required
109	Female	82	6	27	8	76	42	Survivor	Required	Required
110	Female	86	6	33	12	51	38	Non-survivor	Not Required	Required
111	Male	68	6	19	6	46	37	Non-survivor	Required	Required
112	Male	60	6	26	4	46	22	Survivor	Not Required	Required
113	Female	65	4	19	6	42	33	Survivor	Not Required	Required
114	Female	92	7	22	5	39	19	Survivor	Not Required	Required
115	Male	76	7	23	8	65	40	Non-survivor	Required	Required
116	Male	68	4	14	7	69	37	Non-survivor	Required	Required
117	Male	77	11	33	10	70	42	Non-survivor	Not Required	Required
118	Male	61	4	19	5	39	22	Survivor	Not Required	Required
119	Male	67	4	18	4	55	50	Non-survivor	Required	Required
120	Male	63	6	22	4	50	24	Survivor	Not Required	Required
121	Male	90	7	22	12	61	48	Non-survivor	Required	Required
122	Male	65	4	27	5	45	33	Survivor	Not Required	Required
123	Female	54	5	32	7	49	39	Non-survivor	Required	Required
124	Female	84	8	33	9	76	47	Non-survivor	Required	Not Required
125	Female	86	13	13	10	55	41	Non-survivor	Required	Required
126	Male	88	6	31	8	65	47	Non-survivor	Not Required	Required
127	Male	82	10	38	11	83	36	Non-survivor	Not Required	Required
128	Female	80	6	11	4	53	24	Survivor	Not Required	Required
129	Male	88	6	29	10	56	30	Non-survivor	Not Required	Required
130	Male	60	4	18	6	52	31	Survivor	Not Required	Required
131	Male	53	5	37	14	57	39	Non-survivor	Required	Required

132	Male	62	7	36	10	74	37	Non-survivor	Not Required	Required
133	Female	92	7	28	9	67	30	Non-survivor	Required	Required
134	Female	23	2	21	6	27	11	Survivor	Not Required	Required
135	Female	78	8	26	7	46	35	Non-survivor	Required	Required
136	Male	81	12	35	11	82	42	Non-survivor	Required	Required
137	Male	83	6	22	5	61	21	Non-survivor	Not Required	Required
138	Male	66	10	39	16	67	22	Non-survivor	Not Required	Required
139	Male	87	10	41	15	82	46	Non-survivor	Required	Required
140	Male	66	10	19	6	88	41	Non-survivor	Required	Required
141	Female	88	6	25	5	48	29	Survivor	Required	Required
142	Male	58	5	19	5	50	20	Survivor	Not Required	Required
143	Male	62	6	29	9	86	51	Non-survivor	Required	Not Required
144	Male	69	8	40	12	73	40	Non-survivor	Not Required	Required
145	Male	85	11	31	10	89	53	Non-survivor	Required	Required
146	Male	78	7	39	13	58	32	Non-survivor	Not Required	Not Required
147	Male	70	7	23	7	59	42	Non-survivor	Not Required	Required
148	Female	92	10	20	7	68	47	Non-survivor	Required	Required
149	Female	87	10	28	6	64	40	Non-survivor	Required	Required
150	Female	84	9	38	11	80	53	Non-survivor	Required	Not Required
151	Male	85	12	31	10	67	40	Non-survivor	Required	Required
152	Male	70	5	22	5	47	21	Survivor	Not Required	Required
153	Female	39	8	35	9	64	41	Non-survivor	Required	Required
154	Male	63	4	22	6	53	21	Survivor	Not Required	Required
155	Male	56	4	19	9	63	41	Non-survivor	Not Required	Required
156	Male	72	8	17	8	47	21	Survivor	Not Required	Required
157	Female	55	5	23	8	44	20	Survivor	Not Required	Required
158	Male	74	7	36	10	62	25	Non-survivor	Not Required	Required
159	Male	48	2	23	7	44	27	Survivor	Not Required	Required
160	Female	78	10	18	7	60	32	Non-survivor	Required	Required
161	Male	80	7	24	7	45	23	Non-survivor	Not Required	Required
162	Male	62	10	21	5	56	36	Non-survivor	Required	Required
163	Female	79	13	33	12	70	48	Non-survivor	Required	Required
164	Male	26	2	14	5	33	15	Survivor	Not Required	Required
165	Male	27	3	26	8	31	25	Survivor	Not Required	Required

Patients were included in the study after being diagnosed with sepsis or septic shock.
